# Uncoupling protein 2 downregulation by hypoxia through repression of peroxisome proliferator-activated receptor γ promotes chemoresistance of non-small cell lung cancer

**DOI:** 10.18632/oncotarget.14097

**Published:** 2016-12-22

**Authors:** Mingxing Wang, Guoyin Li, Zhiwei Yang, Lei Wang, Lei Zhang, Ting Wang, Yimeng Zhang, Shengli Zhang, Yong Han, Lintao Jia

**Affiliations:** ^1^ Department of Thoracic Surgery, Tangdu Hospital, Fourth Military Medical University, Xi'an, China; ^2^ State Key Laboratory of Cancer Biology, Department of Biochemistry and Molecular Biology, Fourth Military Medical University, Xi'an, China; ^3^ Department of Applied Physics, Xi’an Jiaotong University, Xi’an, China

**Keywords:** uncoupling protein 2 (UCP2), non-small cell lung cancer (NSCLC), chemotherapeutic resistance, hypoxia, PPAR-γ

## Abstract

Hypoxic microenvironment is critically involved in the response of non-small cell lung cancer (NSCLC) to chemotherapy, the mechanisms of which remain largely unknown. Here, we found that NSCLC patients exhibited increased chemotherapeutic resistance when complicated by chronic obstructive pulmonary disease (COPD), a critical cause of chronic hypoxemia. The downregulation of uncoupling protein 2 (UCP2), which is attributed to hypoxia-inducible factor 1 (HIF-1)-mediated suppression of the transcriptional factor peroxisome proliferator-activated receptor γ (PPARγ), was involved in NSCLC chemoresistance, and predicted a poor survival rate of patients receiving routine chemotherapy. UCP2 suppression induced reactive oxygen species production and upregulation of the ABC transporter protein ABCG2, which leads to chemoresistance by promoting drug efflux. UCP2 downregulation also altered metabolic rates as shown by elevated glucose uptake and reduced oxygen consumption. These data suggest that UCP2 is a key mediator of hypoxia-triggered chemoresistance of NSCLCs, which can be potentially targeted in clinical treatment of chemo-refractory NSCLCs.

## INTRODUCTION

Non-small cell lung cancer (NSCLC), which is among the leading causes of cancer mortality worldwide, is characterized by relative insensitivity to radiation and chemotherapy [1]. Most chemotherapeutic drugs block malignant cell survival and proliferation by inducing oxidative damage, impairing DNA biosynthesis, or disrupting cytoskeleton assembly, thereby inducing cell cycle arrest and apoptotic cell death [1]. However, cancer cells can develop resistance to the cytotoxicity of chemotherapy through diverse mechanisms that remain poorly understood [2, 3]. Chemoresistant cells are characterized by increased activity of specific growth factor pathways or constitutive activation of downstream kinases involved in proliferative or survival signaling, defects in the apoptotic machinery, and the overexpression of membrane transporters, e.g., ATP-binding cassette (ABC) transporters, which function as outward pumps for chemotherapeutic drugs [2, 3]. In addition, the hypoxic microenvironment of solid tumors contributes to the development of chemotherapy resistance by promoting malignant phenotypes [4]. The hallmark of hypoxia in neoplastic cells is the expression of hypoxia-inducible factor (HIF). The highly regulated HIF-1α or HIF-2α subunit heterodimerizes with a constitutively expressed HIF-1β subunit to form the active transcription factor HIF-1 or HIF-2, respectively [5]. As a master regulator of oxygen homeostasis, HIF-1 induces the expression of hypoxia-responsive genes involved in the survival and mitosis of carcinoma cells. Meanwhile, HIF-1 is required for the maintenance of malignant phenotypes, such as the self-renewal of cancer stem cells and resistance to anticancer therapeutics [6]. However, the key mediators and cellular events associated with hypoxia-induced chemotherapy resistance remain to be defined.

Uncoupling proteins (UCPs) are a family of mitochondrial proteins, which were originally reported to play essential roles in reducing the mitochondrial membrane potential and reactive oxygen species (ROS) production given the extreme sensitivity of ·O_2_^−^ production to the proton motive force in the mitochondrial matrix of mammalian cells [7]. However, recent studies have proposed that UCP2 reduces ROS by acting as a metabolic regulator of glucose, fatty acid and glutamine oxidation rather than via proton leakage [8]. UCP1 and UCP3 are expressed exclusively in brown adipose tissue and muscle, respectively, whereas UCP2 is found in many types of tissues with particularly robust expression in the lung epithelium and in macrophages [7, 9]. Disruption of UCP2 in mice triggers immune resistance to infection, suppresses inflammatory responses, and results in increased susceptibility to atherosclerosis, which are at least partially attributed to increased ROS production [10]. UCP2 plays a role in carcinogenesis in various tissues and regulates the responsiveness of carcinomas to chemotherapy [9, 11, 12]. However, the precise role of UCP2 in the chemotherapy sensitivity of tumors including NSCLC is currently under debate [9, 12]. In the present study, we show that UCP2 is downregulated in hypoxic NSCLC cells in correlation with reduced susceptibility to chemotherapeutic drugs. NSCLC patients with concurrent chronic obstructive pulmonary disease (COPD) showed increased resistance to chemotherapy. Hypoxia decreased UCP2 via HIF-1-mediated suppression of peroxisome proliferator-activated receptor-γ (PPAR-γ). UCP2 downregulation resulted in increased expression of the ABC transporter ABCG2, as well as reprogrammed glucose metabolism.

## RESULTS

### Hypoxic exposure promotes the resistance of NSCLC cells to chemotherapy

**Figure 1:**
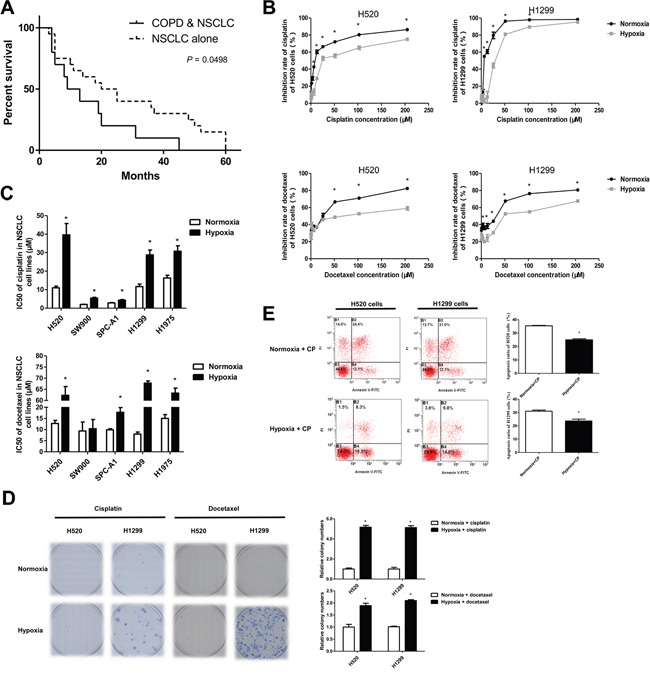
Hypoxia facilitated the development of chemotherapeutic resistance in NSCLCs **A**. Survival of patients with non-small cell lung cancer (NSCLC) alone (n=20) or NSCLC combined with chronic obstructive pulmonary disease (COPD, n=10). **B**. CCK8 assays for NSCLC cells exposed to normoxia or hypoxia (3% O_2_) and treated with the indicated doses of cisplatin or docetaxel. The inhibitory rate was calculated by comparing drug treated cells with the time-matched untreated group. **C**. IC50 values in CCK8 assays of NSCLC cells exposed to normoxia or hypoxia. **D**. Colony formation assays for NSCLC cells treated with cisplatin (2 μM) or docetaxel (20 nM) under normoxia or hypoxia. **E**. Cells treated with cisplatin (30 μM) under normoxia or hypoxia were subjected to FITC-A/PerCP-cy5-5-A staining and flow cytometry assays. Data are represented as the mean ± SEM of n = 3 replicates or representative of 3 independent experiments (B-E). *P < 0.05.

COPD is among the common causes of chronic hypoxemia [13]. Clinical assessment showed that concurrent COPD decreased the survival of NSCLC patients receiving routine chemotherapy, suggesting that hypoxia may facilitate the development of chemoresistance in NSCLCs (Figure 1A). We next examined the effects of hypoxia on the sensitivity of NSCLC cell lines to chemotherapy. Hypoxia alone markedly reduced the inhibitory rate of chemotherapeutic agents on NSCLC cells, whereas exposure of cells to low environmental oxygen increased the cisplatin and docetaxel concentrations causing 50% growth inhibition (IC50) (Figure 1B, 1C). Exposure to hypoxic conditions reduced the ability of chemotherapeutic drugs to inhibit colony formation or trigger apoptosis of NSCLC cell lines H520 and H1299 (Figure 1D, 1E). Taken together, these findings suggest that hypoxia contributes to the development of chemoresistance in NSCLC cells.

### UCP2 downregulation is involved in hypoxia-driven chemoresistance

**Figure 2:**
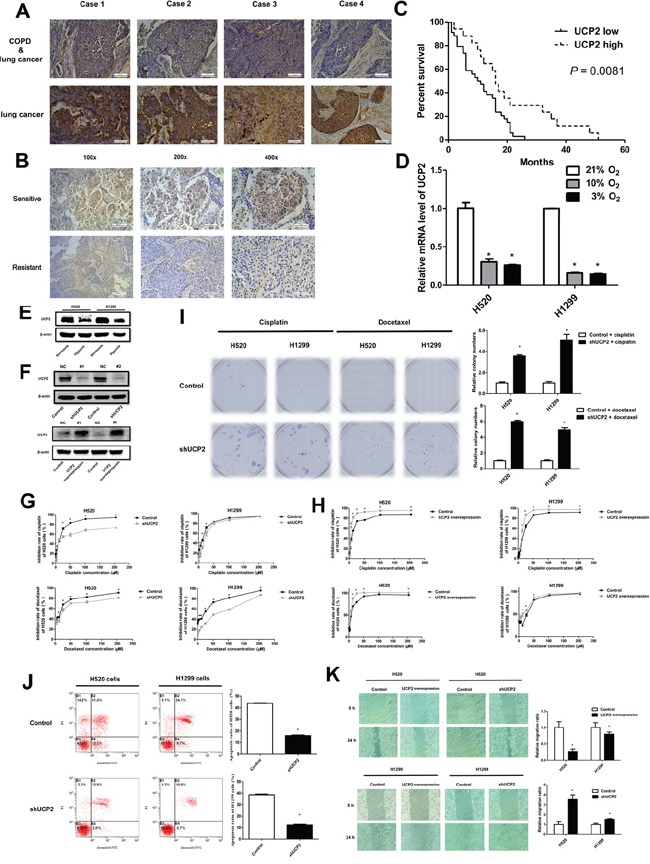
UCP2 downregulation contributed to NSCLC cell chemoresistance in hypoxia **A**. Representative UCP2 immunostaining of tumors from paired patients with NSCLC alone and NSCLC combined with COPD. Magnification, 200×; Scale bars, 100 μm. **B**. Representative immunohistochemical staining for UCP2 using carcinoma tissues from paired chemotherapy-sensitive and -resistant patients. Scale bars, 200μm (100×), 100 μm (200×) and 50μm (400×). **C**. Patients were grouped according to the expression of UCP2 in the carcinomas, and subjected to follow-up investigations. The percent of surviving patients was plotted. **D**. qRT-PCR assays of UCP2 levels in NSCLC cells exposed to the indicated concentrations of oxygen. **E**. Western blot analysis of NSCLC cells exposed to normoxia or hypoxia. **F**. Western blot analysis of cells transfected with UCP2-targeted or scrambled (NC) siRNAs or cells stably transfected with a control or UCP2-overexpressing construct. **G, H**. CCK8 assays for NSCLC cells infected with lentiviruses expressing control or UCP2-targeted shRNAs (G) or cells stably transfected with a control or UCP2-overexpressing construct (H). **I**. Colony formation assays for cisplatin (2 μM) or docetaxel (20 nM) treated NSCLC cells, which were infected with lentiviruses expressing control or UCP2-targeted shRNAs. **J**. Cisplatin (30 μM) induced apoptosis were investigated in NSCLC cells infected with lentiviruses expressing control or UCP2-targeted shRNAs with Annexin V/PI staining kit by flow cytometry assays. **K**. Cell scratch assays for NSCLC cells infected with lentiviruses expressing control or UCP2-targeted shRNAs. Unless indicated, cells were incubated and subjected to drug treatment in normoxia. Scale bars, 100 μm. Data are represented as the mean ± SEM of n = 3 replicates or representative of 3 independent experiments (A-J). *P < 0.05.

UCP2 plays a role in the regulation of cell survival by affecting ROS generation, redox status, and ATP production [9, 11]; therefore, we examined whether UCP2 is involved in the hypoxia-mediated desensitization of NSCLC cells to chemotherapy. Our results showed that UCP2 expression was downregulated in malignant tissues of patients with COPD and NSCLC compared with that in patients with NSCLC alone, suggesting a role of COPD-related hypoxia in regulating UCP2 expression (Figure 2A). Immunohistochemical assessment of clinical samples showed that UCP2 expression was significantly lower in the tumor tissues of chemoresistant NSCLC patients than in those of chemotherapy-sensitive patients (Figure 2B). When patients were grouped according to UCP2 expression in carcinoma tissues, NSCLC patients with high UCP2 expression levels had longer progression-free survival (PFS) than those with low UCP2 expression (Figure 2C). In cultured NSCLC cells, hypoxia downregulated UCP2 in a dose-dependent manner (Figure 2D, 2E). Knockdown of UCP2 in NSCLC cell lines reduced the tumor inhibition rates of chemotherapeutic agents, whereas overexpression of UCP2 sensitized refractory cell lines to chemotherapy (Figure 2F to 2H). UCP2 knockdown promoted *in vitro* colony formation and decreased the rate of apoptosis in NSCLC cells treated with cisplatin or docetaxel (Figure 2I, 2J). In addition, UCP2 silencing accelerated NSCLC migration in a wound-healing assay (Figure 2K). These results suggested that UCP2 plays a regulatory role in the hypoxia-induced resistance of NSCLCs to chemotherapy.

### Hypoxia represses UCP2 expression by downregulating PPAR-γ in NSCLC

**Figure 3:**
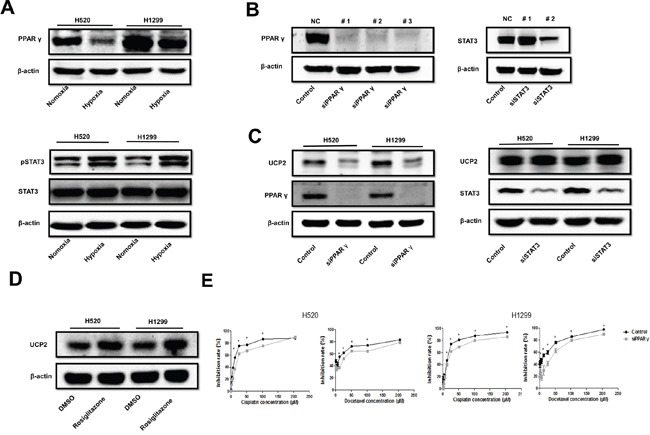
Hypoxia suppressed UCP2 via downregulation of PPAR-γ in NSCLCs **A**. Western blot analysis of cells pre-exposed to normoxia or hypoxia. **B**. Western blot analysis of cells transfected with siRNAs targeting different sites of the indicated mRNAs. The siRNAs with the highest silencing efficacy (#1 for PPAR-γ and #2 for STAT3) were used thereafter. **C**. Western blot analysis of cells transfected with scrambled siRNA or siRNA targeted to the indicated mRNAs. **D**. Western blot analysis of cells treated with vehicle or 4 μM rosiglitazone. **E**. CCK8 assays of cells transfected with scrambled or PPAR-γ-targeted siRNA. Data are represented as the mean ± SEM of n = 3 replicates or representative of 3 independent experiments. *P < 0.05.

Because UCP2 transcription is activated by certain factors such as JAK2/STAT3 and PPARγ [14, 15], we examined the effect of hypoxia on these transcription factors. PPARγ was downregulated while STAT3 phosphorylation was promoted by low oxygen concentrations (Figure 3A). Knockdown of PPARγ but not STAT3 under conditions of normoxia downregulated UCP2, whereas treatment of cells with rosiglitazone, a PPARγ agonist, promoted UCP2 expression (Figure 3B to 3D). PPARγ silencing mitigated the inhibitory potency of cisplatin and docetaxel on NSCLC cells (Figure 3E). While we failed to detect the direct association of PPARγ with the UCP2 promoter (data not shown), these results are in agreement with a previous report showing that PPARγ promotes UCP2 expression by binding to intron 1 of the *UCP3* gene and thereby interacting with the *UCP2* gene via DNA looping [15]. These data suggest that UCP2 is a transcriptional target of PPAR-γ, which was repressed by hypoxia in NSCLC cells.

### HIF-1 mediates the effect of hypoxia on the downregulation of PPARγ and UCP2

**Figure 4:**
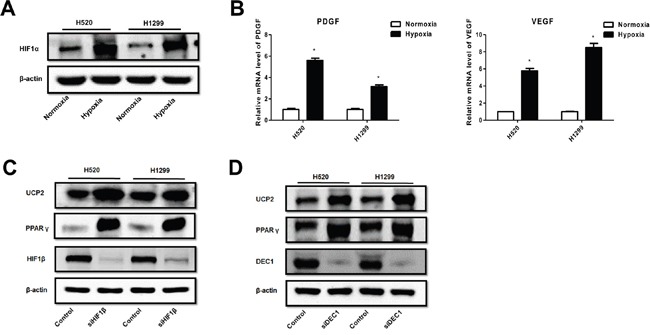
Hypoxia suppression of PPAR-γ and UCP2 was mediated by HIF-1α in NSCLC cells **A**. Western blot analysis of cells pre-exposed to normoxia or hypoxia. **B**. qRT-PCR assays for the expression of HIF-1 target genes. **C, D**. Cells were transfected with siRNAs against the indicated genes, exposed to hypoxia, and subjected to Western blot analysis. Data are represented as the mean ± SEM of n = 3 replicates or representative of 3 independent experiments. *P < 0.05.

HIF-1 is the predominant hypoxia-responsive protein leading to changes in gene expression profiles [16]. Our results showed that hypoxia upregulated the alpha subunit of HIF-1 (HIF-1α) (Figure 4A), consistent with the upregulation of canonical HIF-1 target genes such as VEGF and PDGF-B (Figure 4B) in NSCLC cells. Knockdown of the β subunit of HIF-1, which inhibits HIF-1 transactivation activity, resulted in the upregulation of PPARγ and UCP2 in NSCLC cells (Figure 4C). HIF-1 regulates PPARγ via the transcription factor DEC1 (Stra13) [17]. Consistently, we found that knockdown of DEC1 abrogated the inhibitory effect of hypoxia on PPARγ and UCP2 (Figure 4D). These data suggest that HIF-1-mediated UCP2 suppression is dependent on the downregulation of PPARγ via the DEC1/Stra13 transcriptional repressor complex in NSCLC cells.

### Hypoxia/UCP2-related chemoresistance involves ROS/Nrf2-mediated ABCG2 upregulation

**Figure 5:**
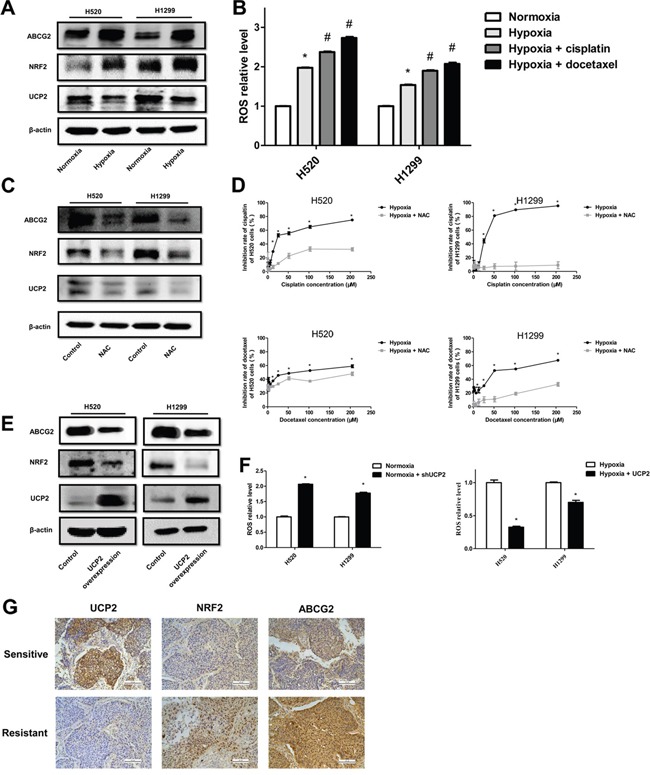
UCP2 suppression induced ABCG2 expression via ROS/Nrf2 in hypoxia-triggered NSCLC chemoresistance **A**. Western blot analysis of cells infected with lentiviruses expressing control or UCP2-targeted shRNAs in normoxia. **B**. Measurement of ROS levels in cells exposed to normoxia or hypoxia and treated with cisplatin (CP, 30 μM) or docetaxel (DTC, 10 μM). *, #P < 0.05 compared with normoxia and hypoxia groups, respectively. **C**. Western blot analysis of cells exposed to hypoxia and treated with vehicle or NAC (10 mM). **D**. CCK8 assays of the cytotoxicity of chemotherapeutic reagents in cells exposed to hypoxia and treated with vehicle or NAC. **E**. Western blot analysis of cells stably transfected with a control or UCP2-overexpressing construct after exposure to hypoxia. **F**. Assays for ROS levels in cells stably transfected with a control or UCP2-overexpressing construct after exposure to normoxia or hypoxia. **G**. Representative immunohistochemical staining using carcinoma tissues from chemotherapy-sensitive and -resistant patients. Scale bars, 100 μm. Data are represented as the mean ± SEM of n = 3 replicates or representative of 3 independent experiments. *P < 0.05.

The sensitivity of malignant cells to chemotherapy is also determined by a class of transmembrane proteins that function as ATP-driven efflux pumps for anticancer agents [18]. ABC transporters translocate various substrates across cellular membranes [18]. Therefore, we examined the expression of ABC transporter members in NSCLC cells, and found that ABCG2 was upregulated upon hypoxia exposure (Figure 5A). ABCG2 can be transcriptionally activated by nuclear factor erythroid 2 p45-related factor 2 (Nrf2), a transcription factor stabilized by ROS [19]. Consistently, we found that Nrf2 expression was enhanced by hypoxic exposure of NSCLC cells, which is in accordance with increased ROS levels and downregulated UCP2 in hypoxic cells (Figure 5A, 5B). Treatment of cells with N-acetyl-L-cysteine (NAC), a ROS scavenger, downregulated Nrf2 and ABCG2 but not UCP2 in hypoxic cells, and improved the sensitivity of cells to cisplatin (Figure 5C, 5D). UCP2 overexpression downregulated Nrf2 and ABCG2, which is consistent with UCP2 suppression of hypoxia-induced ROS production and an increase of ROS levels upon UCP2 knockdown in normoxic cells (Figure 5E, 5F). The correlation of UCP2 downregulation and the increase in Nrf2 and ABCG2 levels with chemotherapy responsiveness was verified by assays using chemo-sensitive and -resistant clinical NSCLC samples (Figure 5G). These data indicated that UCP2 dysfunction may confer chemoresistance through ROS-mediated Nrf2 stabilization and the consequent upregulation of the drug efflux transporter ABCG2 in NSCLC cells.

### UCP2 downregulation promotes metabolic reprogramming of NSCLC cells

**Figure 6:**
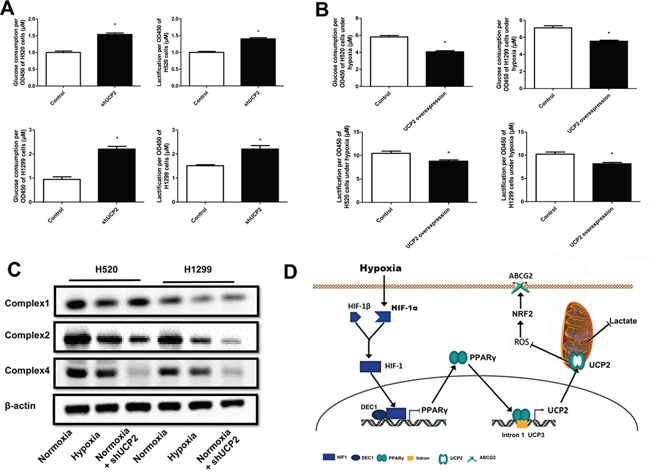
UCP2 downregulation in hypoxia underlies metabolic reprogramming and apoptosis resistance of NSCLC cells **A, B**. Glucose consumption and lactate production assays of cells infected with lentiviruses expressing control or UCP2-targeted shRNAs in normoxia (A) or cells stably transfected with a control or UCP2-overexpressing construct (B). **C**. Western blot analysis of cells infected with lentiviruses expressing control or UCP2-targeted shRNAs prior to exposure to normoxia or hypoxia. **D**. A working model that summarizes the overall findings of this study. Hypoxia downregulates UCP2 by transcriptionally repressing PPAR-γ, which contributes to chemoresistance of NSCLC cells via ROS/Nrf2-mediated upregulation of ABCG2. Data are represented as the mean ± SEM of n = 3 replicates or representative of 3 independent experiments. *P < 0.05.

UCP2 regulates mitochondrial respiration and metabolism in many neoplastic cells [11]. To determine whether metabolic reprogramming is involved in the accelerated proliferation of UCP2-suppressed cells, we examined the glucose metabolism of cells exposed to different oxygen concentrations. We found that UCP2 knockdown remarkably increased glucose uptake and lactate production (Figure 6A), whereas UCP2 overexpression in cells pre-exposed to hypoxia significantly decreased glucose consumption and lactate production (Figure 6B). In addition, the steady-state amounts of several components of the oxidative phosphorylation complexes, including subunits of the respiratory complexes I, II, and IV, were significantly decreased in hypoxic cells (Figure 6C). These results indicated that UCP2 downregulation may promote glucose metabolic conversion from aerobic oxidation to glycolysis in NSCLC cells.

## DISCUSSION

Hypoxia has been well documented as a driving force for the progression and therapeutic resistance of solid tumors including NSCLCs [4, 20]. Hypoxia modulates the microenvironment and the extracellular matrix to facilitate tumor growth, whereas alterations in gene expression profiles and signaling events in cancer cells *per se* dictate their malignant phenotypes, including chemotherapy resistance [21, 22]. Cancer cells may acquire resistance to chemotherapy through the hypoxia-induced expression of drug-pumping proteins, such as multidrug resistance 1 (MDR1)/p-glycoprotein (P-gp)/ABCB1, a known transcriptional target of HIF-1 [23]. Alternatively, hypoxia indirectly induces drug resistance by promoting malignant behaviors such as cell survival, proliferation, and migration, which occurs through the activation of oncogenic signaling or the impairment of the cell death or differentiation machinery [4, 22]. Consistent with these paradigms, we established here that UCP2 is downregulated in response to decreased environmental oxygen supply in NSCLC cells. UCP2 deficiency facilitated the development of chemoresistance via refractory apoptosis and the reprogramming of glucose metabolism in NSCLC cells exposed to chemotherapy. In addition, UCP2 downregulation promoted the efflux of chemotherapeutic drugs by upregulating ABCG2 (Figure 6D). These data suggest that UCP2 is an essential mediator of NSCLC chemoresistance in the context of oxygen deprivation. Despite the apparent discrepancies concerning the role of UCP2 in carcinogenesis and cancer progression in different types of cells, e.g. UCP2 inhibition of apoptosis in hypoxia, our findings are in accordance with recent reports that UCP2 represses the malignant phenotypes of melanoma, glioma, and pancreatic cancer cells and that UCP2 deficiency mimics the effects of hypoxia in pulmonary hypertension [10, 11, 24, 25]. UCP2 was also shown to downregulate HIF-1, which, together with our finding that HIF-1 mediated hypoxia-triggered silencing of UCP2, suggests a regulatory circuit between UCP2 and HIF-1 determining the phenotypes of hypoxic malignant cells [10]. Although it is unclear how UCP2 deficiency facilitates the migration of NSCLC cells, the production of ROS, which was found to be increased by UCP2 knockdown, has been demonstrated to expedite metastasis of lung cancer [26]. In addition, both cell lines used in this study are p53-deficient, which is critically involved in mitochondrial energy dynamics [27]. It is thus worth further investigation whether p53 cooperates with UCP2 to suppress the malignant phenotypes of NSCLC cells.

UCP2 acts as a negative regulator of ROS generation in the mitochondrion that is predominantly involved in the respiratory electron transport chain and ATP biosynthesis, [8]. Endogenous ROS is produced via multiple mechanisms, in which the incomplete reduction of oxygen and thereby the generation of the superoxide radical (·O_2_^−^) on the mitochondrial membrane represents a major source of ROS [28, 29]. Instead of the originally reported uncoupling activity, Bouillaud linked UCP2 regulation of ROS to its involvement in the transport of four-carbon metabolites [8]. Hypoxia increases ROS generation, and ROS induces HIF-1α accumulation in mammalian cells, although the detailed underlying mechanism remains uncharacterized [30]. In the present study, increased ROS production in hypoxic NSCLC cells was at least partially caused by HIF-1 suppression of PPARγ and consequently the PPARγ target gene, UCP2 [15]. In contrast to accelerated ROS production at complex III of the mitochondrial electron transport chain upon acute exposure of cells to hypoxic conditions, the HIF-1/PPARγ/UCP2 pathway defined here may represent a long-term mode of ROS regulation and maintenance in hypoxic carcinomas [31]. As a double-edged sword regarding carcinogenesis and cancer progression, ROS is detrimental to the normal function of macromolecules and the survival of mammalian cells, including neoplastic cells, whereas ROS can also facilitate cancer progression by eliciting genetic mutations and affecting the expression of oncogenes and tumor suppressors [32, 33]. In addition, ROS is required for the activation of growth factor signaling pathways that drive cell cycle progression, and for chronic inflammation, a major mediator of various cancers [29, 34]. Cancer cells exhibit greater ROS stress than normal cells because of increased metabolic activity and mitochondrial malfunction, and mitochondrial ROS perturbation plays a role in conferring resistance to chemotherapy [33, 35]. We found that excessive ROS promoted chemotherapy resistance in NSCLC cells by upregulating the ABC transporter ABCG2 through a mechanism involving the canonical Nrf2 antioxidant pathway [36]. ROS plays a role in apoptosis resistance and the metabolic reprogramming of malignant cells, which may explain our observation that UCP2 deregulation in the context of hypoxia was associated with suppressed apoptosis, elevated glucose uptake, and reduced oxygen consumption in NSCLC cells [37, 38]. Analysis of the abnormalities of UCP2-disrupted mice provides insight into the functions of this protein in addition to blocking ROS generation [39]. Meanwhile, considering the pivotal role of the mitochondrion in apoptotic signaling and cell metabolism, it is likely that other proteins cooperate with UCP2 in the regulation of these processes [40]. Therefore, detailed mechanistic investigations are needed to unravel the way UCP2 regulates the mitochondrial levels of the respiratory complexes.

As a key regulator of chemotherapy susceptibility in NSCLCs, UCP2 expression is fine-tuned at various levels [41, 42]. Several UCP2 gene polymorphisms are associated with distinct mRNA levels and physiological functions [41]. In addition, UCP2 mRNA is post-transcriptionally regulated by both microRNAs and heterogeneous nuclear ribonucleoprotein K (hnRNP K) [41]. Evidence that UCP2 mRNA levels do not correlate with protein expression suggests that UCP2 is also regulated at the translational level [42–44]. Nonetheless, accumulating data indicate that the transcription of the UCP2 gene modulates the cellular levels of the UCP2 protein. UCP2 gene transcription is potentially activated by specificity protein 1, STAT3, FoxA1, Smad4, PPARs, and sterol response element-binding protein 1 in various types of cells [41, 42]. In addition, nutritional metabolites including fatty acids and glutamate, and hormones such as insulin, leptin, and adiponectin, play important regulatory roles in UCP2 expression in adipose and skeletal muscle [42, 45]. Here, we found in NSCLC cells that hypoxia downregulated UCP2 via HIF-1-mediated suppression of the transcriptional factor PPARγ; however, whether an endogenous activating ligand for PPARγ is needed for UCP2 expression remains unclear. In the present study, we failed to detect a direct occupancy of the PPARγ promoter region. This is consistent with a recent study in which chromatin conformation capture was used to show that PPARγ transactivates UCP2 in adipocytes through a DNA loop encompassing the UCP2 promoter and the first intron of the neighboring *UCP3* loci, where PPARγ is primarily recruited [15]. Taken together, our findings revealed a PPARγ-dependent regulatory mechanism of UCP2 by hypoxia and highlighted the multifaceted role of UCP2 in counteracting the chemotherapy resistance of NSCLCs.

## MATERIALS AND METHODS

### Cell culture

The human lung cancer H520, H1299, SW900, H1975, and SPC-A1 cells were obtained from the Type Culture Collection of the Chinese Academy of Sciences. All cell lines were tested and authenticated by short tandem repeat profiling analysis, and were passaged and used within 6 months after authentication. Cells were maintained in RPMI-1640 medium (Gibco BRL, Gaithersburg, MD, USA) supplemented with 10% fetal calf serum (FCS; Gibco BRL), 2 mM L-glutamine, and antibiotics. Cells were incubated in a humidified atmosphere at 20% O_2_ for the normoxic condition, and at 3% O_2_ unless indicated for hypoxic exposure.

### Quantitative reverse transcriptase PCR (qRT-PCR)

Cells were exposed to normoxia or hypoxia, and/or subjected to treatment with cisplatin or docetaxel for 24h. Total RNA was extracted using RNAiso Plus (Takara, #9109, Kusatsu, Shiga, Japan) according to the manufacturer's instructions. Reverse transcription for gene expression was performed using the PrimeScript™ RT Master Mix (Takara, #RR036A). qRT-PCR was performed using the SYBR Green dye (Takara, #RR820A) according to the manufacturer's protocol. The following paired primers were used: UCP2, 5'-CCCAATGTTGCTCGTAATG-3' and 5'-CCCAAAGGCAGAAGTGAAG-3'; VEGF, 5'-TTGCTGCTCTACCTCCAC-3' and 5'-GATGTCCACC AGGGTCTC-3'; PDGF2, 5'-TCTGCTGCTACCTGC GTCTG-3' and 5'-AGAGTGGGAGCGGGTCAT-3'; and β-actin, 5'-CGGGAAATCGTGCGTGAC-3' and 5'-CAGGAAGGAAGGCTGGAAG-3'.

### siRNA synthesis and transfection

siRNAs were designed and synthesized by GenPharma (Suzhou, China) to target the following sequences of mRNAs: 5'-GACGAGAUACAUGAACUCUGC-3' (#1) and 5'-GCUAAAGUCCGGUUACAGATT-3' (#2) for UCP2, 5'-GGUCAGCAGUCUUCCAUGA-3' for HIF-1β, 5'-GCCCUUCACUACUGUUGAC-3' (#1), 5'-GGAGAAGCUGUUGGCGGAGAU-3' (#2) and 5'-GGCUUCAUGACAAGGGAGUUU-3' (#3) for PPARγ, 5'-GGAGGAGGCAUUCGGAAAGUA-3' (#1) and 5'-GCACAAUCUACGAAGAAUCAA-3' (#2) for STAT3, 5'-CGAAACAGGUCAAGAGAUG-3' for DEC1, and 5'-CGGCAAGCUGACCCUGAAGUUCAU-3' for enhanced green fluorescent protein (EGFP) as a negative control. The TurboFect transfection reagent (Thermo Fisher, Waltham, MA, USA) was used to introduce siRNA into cells according to the manufacturer's instructions.

### Constructs for UCP2 overexpression and knockdown

The *UCP*2 coding sequence was amplified from cDNAs of H520 cells using the following primers: 5'-GCTAGCTAGCAGGAAATCAGCATCAT-3' and 5'-GGATCCTCAGGTCAGCAGCAGGAG-3'. The PCR fragment was cloned to the *Nhe*I/*Bam*HI sites of pcDNA3.1(+). Lentiviral vectors for short hairpin RNAs that target the same sequences with the aforementioned siRNAs for UCP2 (#2) and EGFP, respectively, were purchased from GenPharma.

### CCK8 assay

Cells were plated at a density of 1,000 cells/well in 96-well plates and incubated for 24 h in complete medium. Different concentrations of cisplatin or docetaxel were added into wells and incubated for an additional 24 h under normoxic or hypoxic conditions. At the end of the incubation, viable cells were quantified using the Cell Counting Kit-8 (CCK8) method (7Sea Pharmatech, Shanghai, China). Briefly, 10 μl of CCK-8 and 100 μl of medium were added to each well of an assay. After incubation at 37°C for 3 h, a Bio-Rad iMARK™ microplate reader was used to detect the optical density at 450 nm.

### Western blotting

Cells were exposed to normoxia or hypoxia, and/or subjected to treatment with cisplatin or docetaxel for 24h. Cells were lysed for 20 min in ice-cold RIPA lysis buffer supplemented with 1 mM PMSF and a cocktail of protease inhibitors. Blotting was performed with antibodies against UCP2 (Clone # sc-6526, Santa Cruz, Dallas, USA), PPARγ (Cat. # 2443, Cell Signaling Technology, Boston, USA), DEC1 (Santa Cruz), HIF1α (Cat. # 14179, Cell Signaling), HIF-1β (Cat. # 3414, Cell Signaling), Stat3 (Cat. # 9139, Cell Signaling), phospho-Stat3 (Tyr705, Cat. # 9145, Cell Signaling), and ABCG2 (Cat. # 4477, Cell Signaling). Goat anti-rabbit and goat anti-mouse immunoglobulin horseradish peroxidase-linked F(ab)_2_ fragments from Millipore (Billerica, MA, USA) were used as secondary antibodies.

### Colony formation assay

Cells (200/well) were seeded on a 6 cm dish and cultured in complete medium for 10 days. Cells were fixed with methanol, stained with crystal violet, and washed with PBS three times before fixing and staining. Colonies were washed with ddH_2_O after 30 min of staining. The number and area of colonies were calculated with Image J software 5.0.

### Apoptosis assay

Cells were plated at a density of 2.0 × 10^5^ cells/well in 6-well plates, incubated for 24 h in complete medium, serum starved for 12 h, and then treated as indicated. Complete medium with 30 μM cisplatin was added into wells and incubated for 24 h under normoxia or hypoxia. Flow cytometry was used to detect the percentage of dying cells with the Annexin V-FITC/PI apoptosis detection kit according to the manufacturer's protocol (Beyotime, Shanghai, China). Flow cytometry was performed with the FACScan system using Cell Quest software.

### Cell migration assay

Migration was assessed using cell scratch assays in the different groups. Cells were plated in 6-well plates in 2 ml of complete medium. When a monolayer of cells was formed, a scratch was introduced by scraping the monolayer with a P200 pipette tip. After addition of 2 ml of serum-free medium into each well, cells were allowed to migrate for the indicated times. The distances between the edges of the scratch were measured to quantitatively evaluate cell migration.

### Immunohistochemistry

Immunohistochemical analyses were performed using a streptavidin/peroxidase staining kit (Cat. # SP-9000, Zymed Laboratories, San Diego, CA, USA). The 4 μm paraffin-embedded slices were deparaffinized and rehydrated in a xylene/alcohol gradient. After digestion in a 3 M urea solution for 30 min, the slices were boiled in a Citrate Antigen Retrieval Solution for 10 min in a microwave oven. Next, slices were incubated in 5% H_2_O_2_ for 20 min to block endogenous peroxidase activity, followed by goat serum to block non-specific binding, and then incubated with primary antibodies against UCP2 (Clone # sc-6526, Santa Cruz), ABCG2 (Cat. # 4477, Cell Signaling), or Nrf2 (Cat. # 12721, Cell Signaling) in a moist chamber overnight at 4°C. The next day, slices were incubated in secondary antibodies (Millipore, Billerica) for 30 min at 37°C. Finally, slides were incubated in DAB solution (Cat. # ZLI-9017, Zymed) to detect peroxidase activity and counterstained with hematoxylin. Two independent pathologists assessed the stained slides under a microscope in a double blind system.

### ROS assay, glucose consumption, and lactate production

Cells were exposed to normoxia or hypoxia or subjected to treatment with cisplatin or docetaxel for 24h. ROS was detected by the DCFDA Cellular ROS Detection Assay Kit (Beyotime) according to the manual. The ROS level in each group was quantified according to the fluorescence of DCFDA. The fluorescence of DCFDA was excited with a 488 nm laser and detected at 525 nm using a TECAN GENios Pro (TECAN, Männedorf, Switzerland). Glucose and lactate concentrations in the supernatants were measured with Glucose assay and Lactic Acid assay kits (Jiancheng, China). Glucose consumption was calculated by subtracting the glucose concentration detected from the blank well.

### Patients and follow-up

Samples from ten primary NSCLC patients with COPD or emphysema were collected from the clinical samples bank of Tangdu Hospital of the Fourth Military Medical University (Xi’an) in China starting in 2006. Patients with NSCLC alone or NSCLC combined with COPD according to the available clinical information were selected randomly and double-blindly. UCP2 expression level variation between cancerous and paracancerous tissues was examined in another 51 NSCLC patients selected randomly from the sample bank. The clinical characteristics of patients were obtained from hospital records. Sample collection was performed with informed consent and approved by the Ethics Committee of the Fourth Military Medical University. 34 patients that displayed decreased UCP2 in tumors compared with paracancerous tissues in qRT-PCR assay are included in UCP2-low group, whereas 17 patients showing upregulated UCP2 in tumors are designated as the UCP2-high group. Overall survival (OS) was calculated from the date of surgery until death, or until the date of the last follow-up visit for patients who were still alive.

### Statistical analysis

All data were analyzed using SPSS standard version 19.0 and Graph Pad Prism version 5.0. Data were obtained from three independent experiments and presented as the mean ± standard error. Survival rate was analyzed by the Kaplan-Meier method and compared with the log-rank test. *P* < 0.05 was considered statistically significant.

## Abbreviations

COPD: chronic obstructive pulmonary disease
HIF-1: hypoxia-inducible factor 1
MDR1: multidrug resistance 1
Nrf2: nuclear factor erythroid 2 p45-related factor 2
NSCLC: non-small cell lung cancer
PFS: progression-free survival
PPARγ: peroxisome proliferator-activated receptor γ
ROS: reactive oxygen species
UCP2: uncoupling protein 2
